# IT TAKES TWO TO TANGO: potential novel therapies for autosomal dominant optic atrophy

**DOI:** 10.3389/fopht.2025.1688232

**Published:** 2025-11-05

**Authors:** Ritu Sampige, Lyra E.A. Seaborn, Molly Pluenneke, Annika Jyothi, Sophie Saland, Chisom M. Chinedu-Obi, Caroline Keehn, Andrew G. Lee

**Affiliations:** 1 School of Medicine, Baylor College of Medicine, Houston, TX, United States; 2 McGovern Medical School at the University of Texas at Houston, Houston, TX, United States; 3 Tulane University School of Medicine, New Orleans, LA, United States; 4 Department of Ophthalmology, University of Texas Medical Branch, Galveston, TX, United States; 5 Texas A&M School of Engineering Medicine, Bryan, TX, United States; 6 Department of Ophthalmology, Blanton Eye Institute, Houston Methodist Hospital, Houston, TX, United States; 7 The Houston Methodist Research Institute, Houston Methodist Hospital, Houston, TX, United States; 8 Departments of Ophthalmology, Neurology, and Neurosurgery, Weill Cornell Medicine, New York, NY, United States; 9 University of Texas MD Anderson Cancer Center, Houston, TX, United States; 10 Department of Ophthalmology, The University of Iowa Hospitals and Clinics, Iowa City, IA, United States

**Keywords:** autosomal dominant optic atrophy, Targeted Augmentation of Nuclear Gene Output (TANGO), OPA1, idebenone, review

## Abstract

Autosomal dominant optic atrophy (ADOA) is among the most prevalent inherited optic neuropathies with hallmark symptoms of bilateral, painless, progressive, and typically permanent vision loss over time. ADOA can affect patients’ quality of life with debilitating visual symptoms, and there is a pressing need for effective therapeutics. In this paper, we review the current and future investigational therapies for ADOA, including the use of intravitreal injections of antisense oligonucleotides through Targeted Augmentation of Nuclear Gene Output (TANGO), CRISPR-based therapy, genetic editing, gene replacement approaches, and idebenone, a small-molecule mitochondrial modulator. Additionally, we review clinical trials for ADOA treatment and opportunities for future research on ADOA therapeutics, including the utilization of mitochondria-targeted peptides and antioxidants, NAD+ boosters/metabolic support, mitophagy and fission-fusion modulators, and cell-based regenerative therapy. The use of emerging technology to compensate for OPA1 protein haploinsufficiency provides new and vast avenues for the management of this otherwise vision-altering disease. Increased awareness of therapeutics for ADOA will allow for patient counseling regarding treatment access via clinical trials and for underscoring the importance of genetically testing family members, who may be incidentally identified with ADOA in a timely manner for newly available therapies. While patients with ADOA typically have poor visual prognoses, there are increasing promising therapies with the potential for preserving and improving visual function.

## 1 Introduction

Autosomal dominant optic atrophy (ADOA), also known as Kjer’s syndrome, affects approximately 1 in 30,000 to 1 in 50,000 people globally, with differing prevalence among specific geographic populations. For example, 1 in 10,000 individuals in Denmark have ADOA due in part to a founder effect (1–3). Patients with ADOA typically develop bilateral loss of vision beginning in childhood, often in the first decade of life, that is subsequently progressive and irreversible (1, 4, 5). However, the onset, course, and severity of ADOA is variable (i.e., variable expressivity) (1). Some patients experience loss of vision from birth, while clinically significant symptoms do not occur in others until early childhood or adulthood (1), and some patients with ADOA are asymptomatic. The symptoms and signs of ADOA include decreased visual acuity in both eyes (OU), reduced color vision, visual field deficits (typically central or cecocentral scotomas OU), and eventual optic disc atrophy OU (2, 6–8). While the conventional form of ADOA manifests with optic atrophy and vision loss OU, recent literature has shed light on additional clinical findings (ADOA+). The broader ADOA+ phenotype encompasses other systemic, non-ocular findings including sensorineural hearing loss, progressive external ophthalmoplegia, myopathy, ataxia, and peripheral neuropathy, potentially occurring in the absence of optic atrophy decades after initial vision loss (3). Additionally, ADOA+ has been associated with more severe visual symptoms (3).

ADOA is genetically heterogeneous, meaning that there is variation in the causative genetic insult (4, 9, 10). The most commonly implicated gene, *OPA1*, is located on chromosome 3 (3q28) (9). Among individuals with ADOA, the prevalence of *OPA1* mutations ranges from 60-90% (2, 11, 12). The OPA1 protein, synthesized by the *OPA1* gene, is a mitochondrial GTPase required for mitochondrial homeostasis, including mitochondrial import, fusion and fission, cristae formation and maintenance, respiratory chain complex functioning, and apoptosis (5, 8, 13–16). Mutations in one of the two *OPA1* gene copies lead to inadequate amounts of OPA1 protein generation, known as protein haploinsufficiency (17). Thus, mutation of *OPA1* is associated with inadequate mitochondrial function, leading to apoptosis of retinal ganglion cells (RGCs) and atrophy of the optic disc (8, 18). Additionally, ADOA from *OPA1* mutations may also occur via a dominant-negative effect. For instance, missense mutations in the OPA1 GTPase domain may lead to mutated protein product generation that negatively affects the function of the remaining normal product from the wild-type allele (5, 14, 19). In the dominant-negative variant, OPA1 expression levels are not diminished; instead, there is competition between the protein products of the mutated versus wild-type allele (14). In fact, the dominant-negative variant of OPA1-associated ADOA, from the missense mutation, is associated with more severe multisystem, extraocular symptoms, aligning with the ADOA+ phenotype (5, 14, 19, 20). The pathogenesis of ADOA from *OPA1* gene mutations represents a complex interplay of loss-of-function and dominant-negative effects. Furthermore, some *OPA1* mutations occur outside the GTPase domain, such as in the BSE α-helix or the GTPase effector domain and may subsequently impair mitochondrial fusion (15, 16).

## 2 Novel therapeutics for ADOA

To counter the progressive, debilitating effects of the *OPA1* gene mutation in ADOA, several therapeutic mechanisms have been proposed (**Table 1**), including the use of antisense oligonucleotides, gene therapy, and idebenone, with additional investigational therapeutics underway (**Table 2**) via *in vivo* studies and clinical trials.

**Table 1:** Summary of current investigational therapies for autosomal dominant optic atrophy (ADOA).

Modality	Candidate	MOA	Delivery	Evidence/stage	Key considerations and limitations	Clinical pros	Clinical cons	Summary
Allele-independent upregulation (TANGO ASOs)	STK-002 (Stoke Therapeutics); PYC-001 (PYC Therapeutics)	ASOs designed to increase expression of functional OPA1 from the healthy allele (splice/translation modulation); aims to restore mitochondrial function in RGCs	Intravitreal ASO	Investigational Preclinical (cells/animals)	Off-target splicing Long-term RGC exposure Non-allele- specific benefit	No risk of new pathologic gene alterations	Potential for other pathologic protein variants Multi-session therapy required General anesthesia required for intravitreal injections in children	TANGO is designed to increase expression of functional nuclear gene product (OPA1) from the wild-type allele, thus compensating for reduced protein levels and restoring mitochondrial function in retinal ganglion cells. - STK-002: A TANGO ASO (by Stoke Therapeutics) that can be used to modulate OPA1 splicing and increase OPA1 mRNA and protein levels in patient-derived cells and animal models. - PYC-001: A cell-penetrating peptide-antisense oligomer conjugate targeting OPA1 mRNA (by PYC Therapeutics) that increases OPA1 protein expression from the healthy allele.
Gene regulation (CRISPRa)	dCas9-VPR (guide RNAs upstream of OPA1 TSS)	Transcriptional activation of endogenous OPA1 to rebalance all isoforms	AAV dual-vector or LNP (investigational)	Preclinical (patient- derived cells)	Vector size constraints Cas9 immunogenicity Specificity of activation.	Highly efficient delivery system	Risk of immunogenicity Multi-session therapy required General anesthesia required for intravitreal injections in children	CRISPR-based Technologies: - CRISPR Activation (CRISPRa): A gene regulation technology that utilizes a catalytically inactive Cas9 and is fused to transcriptional activators and guide RNAs targeted to promoter regions. This enables targeted upregulation of gene expression without introducing permanent genetic alterations or initiating double-stranded breakages in DNA (unlike traditional CRISPR/Cas9 gene editing). - Direct gene editing: This technique utilizes induced pluripotent stem cells (iPSCs) from patients to successfully correct *OPA1* variants.
Gene Therapy (OPA1 replacement) (73, 74)	NFS-05 (Neurophth)	AAV-mediated OPA1 gene replacement to restore RGC mitochondrial fusion/cristae integrity	Intravitreal AAV	Investigational (preclinical/IND - enabling)	Isoform selection and promoter specificity Intraocular AAV immunity Durability and dose	Highly efficient delivery system Single dose required for persistent therapeutic effect	Risk of immunogenicity Cell apoptosis resulting from DNA damage-toxicity Potential for new pathologic phenotypes from off-target gene modifications General anesthesia required for intravitreal injections in children	NFS-05 is an OPA1 gene-replacement therapy (by Neurophth) that uses an adeno-associated viral (AAV) vector carrying functional OPA1, which is delivered to RGCs via intravitreal injection.
Small-molecule mitochondrial modulator	Idebenone (Raxone)	CoQ10 analogue; shuttles electrons and acts as antioxidant to support RGC ATP production	Oral	Approved for LHON Off-label observational use in ADOA	Variable response Optimal treatment duration vs. disease stage Needs controlled ADOA trials	General anesthesia not required for therapeutic delivery in children	Does not address dysfunction from underlying haploinsufficiency Long-term, continuous therapy required Systemic exposure due to non-focal therapeutic delivery Efficacy is dependent on non-modifiable factors Reported gastrointestinal adverse effects	This drug is a synthetic benzoquinone and Coenzyme Q10 analogue that acts as both a mitochondrial electron carrier and as an antioxidant. It is primarily used in the treatment of LHON. Idebenone shows promise for slowing the progression of *OPA1*-ADOA, and may even provide some restoration of visual function by helping to preserve ATP production and reduce oxidative damage to RGCs. Further studies are needed to evaluate the efficacy in ADOA.

TANGO, Targeted augmentation of nuclear gene output; ADOA, autosomal dominant optic atrophy; ASO, Antisense oligonucleotide; RGC, retinal ganglion cell; CRISPRa, CRISPR activation (transcriptional activation w/o DNA); dCas9, nuclease-dead Cas9; OPA1- optic atrophy 1 (mitochondrial dynamin-like GTPase); LNP, lipid nanoparticle; IND, Investigational New Drug (application); COQ10, coenzyme Q10; LHON, Leber hereditary optic neuropathy; PK, pharmacokinetics; PD, pharmacodynamics; SS-31, elamipretide; ROS, reactive oxygen species; NAD^+^, oxidized nicotinamide adenine dinucleotide; Drp1, dynamin-related protein 1; iPSC, induced pluripotent stem cell; LGN, lateral geniculate nucleus.

**Table 2:** Summary of potential investigational therapies for autosomal dominant optic atrophy (ADOA).

Modality	Candidate	MOA	Delivery	Evidence/stage	Key Considerations and limitations
Mitochondria-targeted peptide	Elamipretide (SS-31)	Binds cardiolipin to stabilize ETC supercomplexes and improve bioenergetics	Subcutaneous; intravitreal under study	Investigational (ocular indications, not ADOA-specific)	Ocular PK/PD unknown in ADOA; systemic vs. intraocular delivery strategy
Mitochondria-targeted antioxidants	MitoQ, SkQ1	Targets ROS scavenging within mitochondria to limit RGC oxidative damage	Oral/topical (varies)	Preclinical/early clinical (non-ADOA)	Translatability to OPA1- haploinsufficiency; long- term safety.
NAD+ boosters/metabolic support	Nicotinamide, Nicotinamide riboside	Boost NAD+ to support mitochondrial function and resilience	Oral	Small studies in optic neuropathies/glaucoma; not ADOA-specific	Dosing, durability, and endpoints specific to ADOA needed.
Mitophagy & fission-fusion modulators	Drp1 inhibitors (e.g., Mdivi-1/P110); OPA1 stabilizers (concept)	Rebalance fusion/fission; enhance mitophagy of damaged mitochondria	Systemic/intraocular (preclinical)	Preclinical (models outside ADOA)	Specificity, off-target mitochondrial effects, ocular delivery feasibility.
Cell-based regenerative therapy	iPSC-derived RGCs; Muller glia reprogramming	Replace or rescue dysfunctional RGCs; trophic support	Subretinal/intravitreal (experimental)	Early preclinical	Axonal pathfinding to LGN, synaptogenesis, immune rejection, scalability.
Autophagy Modulators	Everolimus	mTOR kinase inhibitor	Oral/topical	Early preclinical	Testing with larger sample size and long-term safety determinations is necessary.
SARM1-Directed therapies	Gene therapy/CRISPR/Cas9, SARM1 inhibitors, ASOs	SARM1 knockout	Variable	Early preclinical	Therapeutics are not likely to completely inhibit SARM1. Need more data on the dose dependence of RGC destruction with SARM1 activity to develop therapeutics.

### 2.1 TANGO

Targeted Augmentation of Nuclear Gene Output (TANGO) is a novel and evolving therapeutic modality for individuals with ADOA, specifically those with *OPA1* gene mutations (12, 21). TANGO therapy, delivered intravitreally, works by preserving the wild-type (WT) gene’s transcribed products. In ADOA-directed TANGO therapy, the STK-002 compound is the antisense oligonucleotide (ASO) delivered to bind nonsense-mediated decay exons on pre-mRNA transcribed from the WT *APO1* gene. Specifically, this ASO attaches to nonsense-mediated decay exons in the WT pre-mRNA, preventing WT transcribed mRNA from degrading prematurely (12, 21, 22). As a result, WT *OPA1*’s mRNA pre- and post-splice products increase in stability, abundance, and concentration (22). This produces a greater quantity of WT OPA1 protein products compared to baseline. Over time, the higher concentration of stabilized WT mRNA available for translation is greater than the amount of non-stabilized, mutant mRNA able to undergo translation. Consequently, the mutant-OPA1 phenotype seen in OPA1 haploinsufficiency is overcome (12, 21).

While there are currently no Food and Drug Administration (FDA) approved therapeutics for ADOA in the United States, TANGO technology shows promising results in preclinical studies (12, 21, 23, 24). Recent data from Stoke Therapeutics demonstrated, with preclinical primate models, that repeated intravitreal injections of antisense oligonucleotides bilaterally led to a dose-dependent elevation in OPA1 protein levels four weeks after initial injections in Cynomolgus monkeys and persisted eight weeks after injection therapy (23). Additionally, TANGO technology was recently approved for clinical trials, such as the Phase I/II OSPREY study for evaluation of efficacy among patients with confirmed *APO1* mutations (22, 25).

### 2.2 Gene therapies

Beyond traditional gene replacement approaches like NFS-05, which is a type of OPA1 gene-replacement therapy that uses an adeno-associated viral (AAV) vector carrying functional OPA1 for RGCs, clustered regularly interspersed short palindromic repeat (CRISPR)-based technologies offer innovative solutions for addressing OPA1 haploinsufficiency in ADOA. These approaches can be broadly categorized into two strategies: CRISPR activation (CRISPRa), which upregulates OPA1 expression from the wild-type allele, and direct gene editing, which corrects pathogenic mutations at the genomic level.

#### 2.2.1 CRISPR activation

Similar to TANGO, CRISPRa addresses the fundamental problem of OPA1 haploinsufficiency without requiring correction of the underlying mutation; however, CRISPRa accomplishes this by altering gene expressivity. By this mechanism, the Cas9 endonuclease is catalytically inactivated (dCas9) and fused to transcriptional activators (e.g., VP64, p65, Rta), enabling targeted upregulation of gene expression (26). CRISPRa directs guide RNAs upstream of the *OPA1* transcription start site near the *OPA1* promoter, thus enhancing expression across all OPA1 isoforms and potentially restoring physiological protein concentration and mitochondrial function (26).

This approach offers several theoretical advantages over conventional gene replacement therapy. AAV-based gene therapies are limited by viral packaging capacity and typically deliver only a single isoform of *OPA1*. In contrast, CRISPRa leverages the endogenous wild-type *OPA1* gene, thereby restoring physiologic levels of all eight isoforms. Recent preclinical studies showed promising results in patient-derived fibroblasts carrying the common c.2708_2711del OPA1 variant. Using a dCas9-VPR system with guide RNAs positioned ~150–300 bp upstream of the transcription start site, researchers achieved significant upregulation of OPA1 expression (26). Interestingly, isoform 5 expression was selectively increased while maintaining appropriate splicing regulation, suggesting that cells can preserve homeostatic isoform ratios under CRISPRa stimulation (26).

Functionally, CRISPRa treatment improved the classic phenotypes of OPA1 deficiency, including mitochondrial network fragmentation. Treated cells displayed increased summed branch length and network branching, indicating partial restoration of mitochondrial connectivity. Importantly, CRISPRa constructs can be packaged into AAV vectors for potential intravitreal delivery, raising the possibility of clinical translation-like conventional gene replacement, but with the added advantage of restoring physiologic isoform diversity (26).

#### 2.2.2 Direct gene editing

Another potential CRISPR-based therapy involves directly correcting pathogenic *OPA1* mutations. Unlike CRISPRa, which enhances expression from the WT allele, this strategy aims to restore normal gene function by repairing the causative mutation, potentially providing a permanent cure. Studies using patient-derived induced pluripotent stem cells (iPSCs) have shown successful correction of *OPA1* variants. For example, correction of the c.1334A>G (p.R445H) mutation with CRISPR-Cas9 restored mitochondrial morphology, normalized oxidative phosphorylation capacity, stabilized mitochondrial DNA, and improved apoptosis resistance, thus reversing many forms of disease-associated cellular dysfunction (27). Similarly, correction of additional *OPA1* mutations in iPSC-derived retinal ganglion cells improved mitochondrial bioenergetics and reduced disease-associated phenotypes (28). Advances in base-editing and prime-editing technologies also raise the possibility of correcting OPA1 mutations with higher precision and fewer off-target effects, further expanding therapeutic feasibility (29).

### 2.3 Idebenone

In addition to gene-based therapies, novel pharmacologic agents are being explored to slow the progression of ADOA. One such drug is idebenone, a synthetic benzoquinone that has already shown promise for improving visual acuity in patients with Leber hereditary optic neuropathy (LHON).

Similar to ADOA, LHON is a mitochondrial disease that causes RGC dysfunction and apoptosis, generally leading to bilateral central vision loss (30). Furthermore, both LHON and ADOA show similar patterns of optic nerve degeneration, although ADOA typically presents earlier in life while LHON onset is more delayed and progression more rapid (31, 32). However, the mutations responsible for LHON affect genes encoding the NADH:ubiquinone oxidoreductase (complex I) subunit, a component of the mitochondrial oxidative phosphorylation system that generates ATP (33). This subunit is implicated in multiple cell signaling pathways and is thought to be one of the main sites of reactive oxygen species (ROS) production (34). Consequently, pathogenic complex I variants result in reduced ATP formation, increased mitochondrial ROS creation, and, ultimately, cell death.

First synthesized in the 1980s, idebenone was initially investigated for use as a potential treatment of Alzheimer’s disease prior to its utilization for mitochondrial disorders (35, 36). Subsequent investigations delved into its efficacy for managing other conditions. Like other benzoquinones, idebenone serves to transfer electrons within the mitochondrial respiratory chain and has been shown to act as a potent antioxidant (37–40). Though idebenone shares a quinone moiety with Coenzyme Q_10_ (CoQ_10_), a naturally occurring benzoquinone marketed as a dietary supplement to mitigate a range of conditions from migraines to heart failure and more, the two compounds differ in several key ways (41). Notably, the bioactivation of mitochondrial CoQ_10_ is dependent on proper mitochondrial function, whereas idebenone is predominantly activated in the cytoplasm (37). Furthermore, in contrast to CoQ_10_, idebenone can channel electrons beyond the complex I directly to complex III, thus maintaining the production of ATP even in the setting of dysfunctional complex I (38, 42–44). Researchers soon recognized that idebenone’s unique properties could lend potential applications in LHON and other mitochondrial diseases.

In 1992, a case report of LHON successfully treated with idebenone was published in *The Lancet* (45). However, it was not until June 2015 that the European Medicine Agency (EMA) approved idebenone (Raxone, Santhera Pharmaceuticals, Liestal, Switzerland) for use in LHON patients. Currently, it remains the only approved drug for treating visual impairment in adolescents and adults with LHON (46). Even so, evidence for its efficacy in visual recovery remains limited and controversial (35). Recommendations for idebenone use in LHON vary depending on the chronicity of disease (30, 47). Additionally, treatment effects have been shown to differ with patient age, sex, and mtDNA mutation.

Despite the existence of conflicting data, idebenone’s successes inspired trials of the drug in patients with ADOA. A pilot study of seven participants suggested that treatment with idebenone for at least one year may improve visual acuity in *OPA1*-mutant ADOA (32). Similarly, a subsequent cohort examination of 87 *OPA1*-ADOA patients found significantly greater visual stabilization and recovery in patients treated with idebenone compared to untreated subjects, even after controlling for confounders (32). Dosages ranged from 135–675 mg/day, with the majority (48%) of idebenone-treated subjects taking 540 mg/day. Another study of *OPA1*-ADOA patients treated with 900 mg/day for 12 months found significant improvement in visual acuity, visual field testing, and self-reported vision-related quality of life compared to baseline measures. However, there were no observed changes in color vision or contrast sensitivity (48).

Idebenone is generally considered to be safe and well-tolerated, with most clinical studies demonstrating high compliance with medication intake and low or no idebenone-related adverse events (43, 47–49). A longitudinal analysis of idebenone’s efficacy in LHON patients found that, of the adverse events reported, the majority were mild and gastrointestinal in nature (e.g., diarrhea), with the few observed severe/fatal outcomes considered unrelated to idebenone treatment (46).

Overall, idebenone shows promise for slowing the progression of *OPA1*-ADOA and may even restore some visual function by helping to preserve ATP production and reduce RGC oxidative damage. Larger-scale trials and prospective investigations are needed to better elucidate the drug’s efficacy. Additionally, it remains unclear whether idebenone may benefit ADOA patients with non-*OPA1* mutations. Future studies addressing these gaps could determine appropriate treatment candidates and dosing schedules among ADOA patient populations, potentially leading to new breakthroughs in pharmaceutical management.

### 2.4 Therapeutics for dominant-negative *OPA1* mutations

TANGO and CRISPRa techniques may have limited application for patients with dominant-negative *OPA1* mutations compared to the haploinsufficiency form of *OPA1* mutations. More specifically, in dominant-negative *OPA1* mutations, the protein product of the mutated *OPA1* gene negatively affects the function of the protein product of the normal *OPA1* allele, thus generating a more severe ADOA phenotype, including mitochondrial dysfunction and extraocular disease presentation, such as ADOA + (5, 14, 19, 20). TANGO technology aims to increase OPA1 protein generation (12, 21); however, by doing so, both the normal and the mutated OPA1 protein is inadvertently increased. Similarly, while CRISPRa may be successful in genetically editing the mutated copy of the *OPA1* gene (26), any remaining mutated OPA1 protein has the capacity to disturb the function of the normal OPA1 protein, thus perpetuating the ADOA phenotype. Instead, specific unique strategies are necessary to address dominant-negative *OPA1* mutations, such as selective silencing of the mutant allele instead of an overexpression of the wild-type allele. For instance, viral vectors may be considered to block the mutant OPA1 allele (5). Additionally, small interfering RNAs (siRNAs) or ASOs may be harnessed to target certain mRNA sequences for cleavage, degradation, and subsequent selective gene silencing (5).

### 2.5 Other clinical trials for ADOA therapeutics

Currently, there are no other FDA approved treatments for individuals with ADOA. However, various novel therapies have entered preclinical and clinical testing for ADOA in recent years. They are broadly divided into pharmacological neuroprotection, gene therapy, and cell-based regenerative therapies (5).

PYC-001 is an OPA1-targeting ASO developed by PYC Therapeutics. Specifically, PYC-001 is a cell-penetrating peptide-antisense oligomer conjugate targeting OPA1 mRNA (50). In patient-derived retinal cell models, PYC-001 increased OPA1 protein production to nearly normal levels, restoring the mitochondrial network (50).

A Phase 1b open-label, randomized sequential dose trial has been set to assess safety, tolerability, and optimal dosage of PYC-001 intravitreal injection therapy for patients with confirmed *OPA1* mutations. Additional metrics will assess ocular structural and function changes as well as the pharmacokinetics of PYC-001 (51). As of August 2025, the trial has not begun recruiting participants yet.

Gene therapies provide another potential therapeutic option for patients with ADOA. NFS-05, an *OPA1* gene-replacement therapy created by the biotech company Neurophth, uses an adeno-associated viral (AAV) vector carrying functional *OPA1* that is delivered to RGCs via intravitreal injection. This therapy received approval to begin clinical trials in Australia for the treatment of ADOA (52).

Finally, because RGC loss is central to the pathophysiology of ADOA, several trials have explored cell-based neuroprotection. In the Stem Cell Ophthalmology Treatment Study (SCOTS), autologous bone marrow-derived stem cells (BMSCs) were infused into the eyes of ADOA patients. In a report of 6 patients, 5 of 6 (83%) showed improved visual acuity, with an average improvement for all eyes of 29.5% (53). These findings demonstrate another potential treatment. The NIH-registered SCOTS2 trial is currently recruiting participants to evaluate the safety and efficacy of ocular BMSC in retinal and optic nerve damage and disease (54).

Overall, the recent clinical trials investigating potential therapies for ADAO, including TANGO ASO (STK-002 and PYC-001), gene therapies (NFS-05), idebenone, and cell-based regenerative therapies, collectively show promise toward disease-modifying treatment of ADOA.

## 3 Discussion

### 3.1 Challenges associated with novel therapeutics for ADOA

#### 3.1.1 Genetic heterogeneity

The challenges associated with novel therapeutics for ADOA are multifaceted and involve both the biology of the disease and transitional barriers. The first challenge arises from the heterogeneity of *OPA1* mutations. More than 400 pathogenic variants have been described, each producing variable effects on protein function and clinical severity (7). These mutations range from missense and nonsense variants to splice-site changes and large deletions, each producing variable effects on OPA1 protein stability, mitochondrial dynamics, and ultimately clinical severity (5). For example, missense mutation in the GTPase domain, such as the dominant-negative variant of *OPA1*-linked ADOA often results in early-onset, severe optic atrophy, whereas truncating mutations may produce milder or later-onset phenotypes (5, 14). Another example of the genetic heterogeneity of ADOA includes the interplay of mutations within and beyond the GTPase domain (16). In fact, a recent study of patients with ADOA revealed two forms of *OPA1* mutations, including the V465F mutation within the GTPase domain (GTPase β-fold) and the V560F mutation outside of the GTPase domain (BSE α-helix) (16). Another study of ADOA pathogenesis showed an association between the deletion of the GTPase effector domain of OPA1 and ADOA development, apart from GTPase domain missense mutations (55). In fact, mutations in the OPA1 GTPase effector domain have been linked with partial defects in mitochondrial fusion and GTPase functioning (15).

This diversity complicates the design of treatments that can be broadly effective across the patient population, since a therapeutic that rescues haploinsufficiency may not adequately correct dominant-negative variants. In addition, variable expressivity within families carrying the same mutation further emphasizes the need for mutation-specific or genotype-stratified therapeutic approaches, yet such precision medicine strategies remain underdeveloped in ADOA research (7). This genetic heterogeneity; therefore, establishes a fundamental barrier for the design of universal therapeutic strategies.

#### 3.1.2 Retinal ganglion cell delivery

Furthermore, a pivotal aspect requiring attention is efficient delivery to retinal ganglion cells (RGCs), which are the primary site of degeneration in ADOA. RGCs are post-mitotic and situated deep in the inner retina, a compartment that is relatively difficult to access with therapeutic agents, making efficient delivery a challenge. Preclinical studies have shown that intravitreal injection of ASOs and adeno-associated viral (AAV) vectors can achieve a selective degree of uptake into RGCs (23). For instance, AAV2 vectors demonstrate preferential tropism for RGCs following intravitreal delivery, and animal models of optic neuropathies have shown partial preservation of visual function with this approach (24). However, translation to durable widespread expression in human RGCs remains an unmet need. Clinical trials of intravitreal AAV-based therapies in other optic neuropathies, such as Leber hereditary optic neuropathy (LHON), emphasize both the promise and limitation of this route, efficacy has been variable and expression levels often subtherapeutic (24). These findings suggest that while vector-based and oligonucleotide-based therapies are biologically feasible, optimizing delivery methods to achieve long-term RGC transduction without compromising safety is still a major challenge.

#### 3.1.3 Modality-specific limitations

Each therapeutic modality introduces its own set of limitations. ASOs, such as those used in the STK-002 TANGO program, require repeated intravitreal dosing to sustain activity, raising concerns about patient compliance and the cumulative, although low, risks associated with repeated ocular injections, such as endophthalmitis, retinal detachment, and repeat general anesthesia required for intravitreal administration in children (12, 21, 23). In parallel, gene therapy approaches that rely on AAV-mediated OPA1 delivery face the inherent limitation of viral packaging capacity, since the OPA1 gene (approximately 100 kb isoforms) exceeds the optimal size for standard AAV vectors (56). Moreover, systemic or intraocular immune responses to the vector can limit transduction efficiency or necessitate immunosuppression (57). Small-molecule therapies such as idebenone, designed to bypass mitochondrial dysfunction and mitigate oxidative stress, have demonstrated modest clinical benefit in subsets of patients (32). For example, idebenone has been shown to improve visual function in certain patients with optic neuropathies, yet responses in ADOA remain inconsistent, possibly reflecting underlying genetic heterogeneity. Taken together, these examples emphasize how each therapeutic modality presents novel challenges that must be balanced against its potential benefits.

Additionally, despite preclinical results regarding CRISPR-based gene therapies, several translational challenges remain. First, homology-directed repair and other editing mechanisms can be less efficient in non-dividing cells, like retinal ganglion cells, which may limit *in vivo* correction rates. Second, safe and effective delivery of CRISPR constructs into RGCs presents hurdles, including viral packaging constraints, immune response risk, and the need for durable long-term expression. Finally, rigorous *in vivo* studies are needed to address the durability of gene correction and to ensure minimal off-target genomic modifications.

### 3.2 Clinical trial design

Finally, the design and execution of clinical trials in ADOA present significant logistical difficulties. Given the rarity of ADOA, patient recruitment in clinical trials is inherently limited and challenged; hence, most studies to date have been small, non-randomized, open-label, or uncontrolled cohorts, weakening the strength of conclusions that can be drawn (22). For instance, several idebenone studies in ADOA included fewer than 20 patients, with heterogeneous outcome measures, making it difficult to establish reproducible efficacy (48). The lack of standardized endpoints further complicates interpretation, visual acuity and optical coherence tomography (OCT) of the retinal nerve fiber layer (RNFL) are commonly used however may not capture subtle disease progression or early treatment effect (58). Moreover, the slow, variable natural history of ADOA progression necessitates lengthy follow-up periods, increasing trial costs and complexity. These challenges suggest not only the difficulty of generating high-quality evidence but also the urgent need for more coordinated, multicenter trial infrastructure to advance ADOA therapeutics from concept to clinical reality.

### 3.3 Opportunities for further exploration

Future work in ADOA therapeutics is focused on overcoming the challenges outlined above, with the most immediate advances expected from ongoing clinical trials. The OSPREY study, which evaluates the safety and efficacy of STK-002, represents the first controlled human trial of an antisense oligonucleotide (ASO) therapy specifically designed for ADOA (22). Early reports suggest acceptable tolerability and evidence of target engagement; however, longer-term data are needed to confirm efficacy (59, 60). If successful, OSPREY could establish proof-of–concept that modulating OPA1 expression with ASOs is both feasible and clinically meaningful, setting a benchmark for subsequent trials. This would not only validate ASOs as a therapeutic modality in ADOA but may also provide critical insights into trial design, including the feasibility of patient recruitment and utility of different outcome measures in a rare disease context. Positive readouts could therefore catalyze broader investment and accelerate development pipelines across multiple therapeutic platforms.

At the same time, advances in gene editing hold considerable promise for more durable, mutation-specific interventions. Traditional CRISPR-Cas9 approaches face limitations due to double-strand DNA breaks and the risk of off-target mutations. Next-generation platforms, such as base editing and prime editing, offer more precise tools to correct pathogenic *OPA1* variants at the nucleotide level without introducing double-strand breaks (26). Preclinical studies in other monogenic retinal diseases have demonstrated the feasibility of these approaches, showing restoration of normal protein function with fewer off-target effects. Although OPA1’s large size and isoform complexity add unique challenges, the rapid evolution of editing tools increases the likelihood that such approaches could be adapted for ADOA. Importantly, combining gene editing with improved delivery platforms may help overcome barriers to efficient RGC targeting, a key limitation of earlier gene therapy efforts.

Parallel to gene editing, refined delivery methods will improve the efficiency of RGC targeting. Conventional AAV vectors are constrained by their limited packaging capacity and immunogenicity, but engineered capsids with enhanced tropism for RGCs are currently in development (56). In addition, non-viral nanoparticle systems, including lipid nanoparticles (LNPs) have shown increasing promise in ocular drug delivery, offering the potential for reduced immune activation and repeat dosing (57). These approaches could broaden the therapeutic window while providing a modality to deliver larger constructs, including those required for full-length OPA1 isoforms or gene editing machinery. Advances in delivery, therefore, represent a pivotal step toward achieving the durable and widespread expression necessary for long-term disease modification in ADOA.

Combination strategies are also being explored to maximize therapeutic benefit. For example, small molecules such as idebenone could be used (48) alongside gene-based therapies to stabilize mitochondrial function during the early treatment period, potentially mitigating cellular stress while genetic correction takes effect. Such multimodal approaches have precedent in other mitochondrial diseases, where pharmacologic and genetic strategies together enhance durability of response. For instance, the combination use of small-molecule inhibitors, ASOs, and gene-therapy approaches may help to further increase OPA1 protein to correct haploinsufficiency and to improve mitochondrial functioning. However, established, robust data is first necessary regarding each of these therapies individually for ADOA treatment to better understand the effects of potential combination. Further testing of combination strategies is necessary to evaluate safety and efficacy as combination therapy does not necessarily guarantee a synergistic effect and may run the risk of toxicity. Beyond therapeutic efficacy, progress in clinical trial infrastructure will play a crucial role in advancing the field. International registries, harmonized outcome measures, and multicenter natural history studies are beginning to emerge, providing the platform necessary for larger and more rigorous randomized controlled trials (58). As these efforts mature, they will enable a shift from small, underpowered exploratory studies to robust investigations capable of generating regulatory-grade evidence. Collectively, these developments point toward a future in which targeted, durable, and accessible therapies may become available for patients with ADOA.

Other emerging approaches potentially include mitochondrial transplantation, which is an experimental strategy aimed at restoring mitochondrial function. Although mitochondrial transplantation has not yet been used to treat clinical ADOA, the replacement of mutated mitochondrial DNA (mtDNA) with wild-type mtDNA via cybrid technology has displayed correction of another mitochondrial disease: Leber’s hereditary optic neuropathy (61). Additionally, mitochondrial transfer has been shown to improve morbidity and mortality in a murine model of Leigh syndrome, another mitochondrial disease (62). Furthermore, autophagy modulation is another promising treatment method for ADOA. In fact, recent studies in patient-derived skin fibroblasts have shown that OPA1 mutations displaying haploinsufficiency may impair autophagy and trigger cellular senescence (19, 63). Thus, treatment of fibroblasts with everolimus, an mTOR kinase inhibitor, was successfully able to restore these phenotypes, suggesting a potential therapeutic strategy for ADOA (63). More specifically, such evidence showed the ability for everolimus to protect OPA-1 mutated fibroblasts from senescence and to restore autophagy (63). However, such treatment may be more specific to OPA1 haploinsufficiency since dominant-negative variants of OPA1 mutations are contrastingly linked with increased autophagy and mitophagy (19). Additionally, another study revealed a role for autophagy in the control of mitochondrial content in axons and in visual loss in a mouse model of ADOA caused by OPA1 deletion (64). More specifically, the deletion of autophagy and mitophagy genes in RGCs with mutated *OPA1* is linked with a restoration of mitochondrial content and protection from vision loss (64).

*OPA1* mutations are linked with an activation of sterile alpha and TIR motif containing 1 (SARM1), a prodestructive factor that codes for a protein, known as an injury-driven NADase, that breaks down NAD^+^ into cyclic ADP-ribose (65). SARM1 has a sequence that allows for specific mitochondrial targeting and is activated in degenerative, including neurodegenerative, states, leading to axon degeneration and neuron death (65). A recent study demonstrated that SARM1 knockout mitigates degeneration in an ADOA mouse model (65). In fact, emerging SARM1-directed therapies, such as small-molecule SARM1 inhibitors, ASOs, and gene-therapy approaches that target SARM1), may be applied to the treatment of ADOA due to the role of SARM1 activation in ADOA pathogenesis (65). Current emerging research shows how gene therapy may be harnessed to block SARM1 and the downstream axon degeneration (66), how SARM1 inhibitors can help restore axons that are in moderate injury prior to full degradation (67), and how *Sarm1* ASOs can reduce SARM1 levels and impede axon degeneration (68), thus showing potential for potential use in ADOA therapeutics. Furthermore, spliceosome-mediated RNA trans-splicing may be further investigated as a potential therapeutic approach for ADOA. This form of gene therapy targets mutations at the level of transcription (69), which may be harnessed for OPA1 replacement or to block SARM1. For example, a recent study aimed to fix *OPA1*-associated splice mutations to address haploinsufficiency with engineered U1 snRNA and showed that engineering the U1 binding process can address the *OPA1* splice defect and subsequently elevate the level of properly spliced *OPA1* transcripts (70). Lastly, retinal or retinal ganglion cell organoids derived from patient-induced pluripotent stem cells with OPA1 mutations have great potential as a platform for future therapy testing, given their ability to recapitulate disease-relevant phenotypes *in vitro* (71, 72).

## 4 Conclusion

Although ADOA is a rare genetic disease, it is one of the most common hereditary optic neuropathies and can drastically impact patient quality of life. The increasing use of novel therapies – such as TANGO technology, CRISPR gene editing, and gene replacement therapy to address the OPA1 protein haploinsufficiency; idebenone to stall RGC destruction and protect the Retinal Nerve Fiber Layer; cell-based regenerative therapy to replace dysfunctional RGCs; and targeted mitochondrial peptides and antioxidants, NAD+ boosters, and mitophagy modulators for mitochondrial support – is creating exciting opportunities for the management of this vision-threatening condition.

Ophthalmologists and patients should be aware of these options and the availability of clinical trials for ADOA. Increased knowledge of such therapeutics will open doors for patient counseling regarding interventions and the importance of referring family members for genetic testing. Both will enable pathogenic mutation identification in a timely manner and allow early treatment intervention. In short, research into these therapeutic modalities for hereditary optic neuropathies is ongoing and represents a major advance in ophthalmology.
